# miR‐181a/b therapy in lung cancer: reality or myth?

**DOI:** 10.1002/1878-0261.12420

**Published:** 2019-01-03

**Authors:** Cornelia Braicu, Diana Gulei, Roxana Cojocneanu, Lajos Raduly, Ancuta Jurj, Erik Knutsen, George Adrian Calin, Ioana Berindan‐Neagoe

**Affiliations:** ^1^ Research Center for Functional Genomics, Biomedicine and Translational Medicine ‘Iuliu Hatieganu’ University of Medicine and Pharmacy Cluj‐Napoca Romania; ^2^ MedFuture Research Center for Advanced Medicine ‘Iuliu Hatieganu’ University of Medicine and Pharmacy Cluj‐Napoca Romania; ^3^ Department of Experimental Therapeutics The University of Texas MD Anderson Cancer Center Houston TX USA; ^4^ Center for RNA Inference and Non‐Coding RNA The University of Texas MD Anderson Cancer Center Houston TX USA; ^5^ Department of Functional Genomics and Experimental Pathology The Oncology Institute ‘Prof. Dr. Ion Chiricuta’ Cluj‐Napoca Romania

**Keywords:** lung cancer, miR‐181a/b, therapy

## Abstract

Despite substantial progress in oncology, lung cancer remains the number one malignancy in terms of both incidence and mortality rates, and there thus remains an urgent need for new therapeutic alternatives. MicroRNA (miRNA) have an important role in cancer initiation and progression due to their capacity to interfere with transcriptional signaling and regulate key cellular processes. miR‐181a and miR‐181b (miR‐181a/b), which are located on chromosomes 1 and 9, are pathologically expressed in the tumor tissue and plasma of patients diagnosed with lung cancer. The miR‐181a/b regulatory mechanisms are sophisticated and are directly related to different target genes. In recent years, an ever‐increasing number of studies have focused on the biological relevance of miR‐181a/b in key cellular processes. In this paper, we aim to discuss the challenging experimental data related to miR‐181a/b and their potential use for the development of new therapeutic approaches in lung cancer. We will further present the ongoing issues regarding the regulation of their multiple target genes, and their potential use as biomarkers and therapeutic targets in this deadly malignancy.

**Abbreviations**
A549/cis: A549 cell line resistant to cisplatin
ADC: adenocarcinoma
AMO‐miR‐181: *anti*miR‐181a oligonucleotides
EMT: epithelial‐to‐mesenchymal transition
IL: interleukin
LNA: locked nucleic acid
lncRNA: long ncRNA
LSCC: lung squamous cell carcinoma
MAK: mitogen activated protein kinases
miRNA: microRNA
ncRNA: non‐coding RNA
NF‐κB: nuclear factor kappa beta
N: normal tissue
NSCLC: non‐small cell lung cancer
SCLC: small cell lung cancer
siRNA: small interfering RNA
TGFβR1: transforming growth factor β receptor 1
TGFβR2: transforming growth factor β receptor 2
TGFβ: transforming growth factor β
TNM: tumor; node; metastases
T: tumor tissue

## 1 Introduction

Lung cancer is the most frequent cause of death for patients diagnosed with cancer worldwide, and is responsible for approximately 18.4% of the total cancer deaths in both sexes (Bray *et al*., 2018; Didkowska *et al*., 2016). The mortality and incidence ratios in both developed and developing countries (Bray *et al*., 2018) are affected by the presence of various risk factors, the efficiency of the diagnostic methods, and/or the treatment accessibility (Bray *et al*., 2018; Choi *et al*., 2017). There are two histological subtypes of lung cancer: small cell lung cancer (SCLC, 15%) and non‐SCLC (NSCLC, 85%) (Herbst *et al*., 2018; Liu *et al*., 2016). The most common NSCLC subtypes are lung adenocarcinoma (ADC) and squamous cell carcinoma (SCC; Herbst *et al*., 2018).

Lung cancer progression is dependent on the tumor microenvironment and is caused by different factors (tobacco smoke remains the most relevant, along with asbestos, arsenic, inorganic arsenic compounds, and ionizing radiation; Field and Withers, 2012) which affect the clinical phenotype, the development of bone and brain metastases, and the response to therapy (Herbst *et al*., 2018; Popper, 2016). One major clinical issue is the lack of early diagnostic and prognostic markers, together with the absence of specific treatment targets. Therefore, advanced forms of disease are usually unresponsive to chemotherapy and only 10–15% of patients have a survival rate of over 5 years. The use of checkpoint inhibitors improved the response rate and survival of some lung cancer patients (Aguiar *et al*., 2017; Jain *et al*., 2018; Thungappa *et al*., 2017). It is therefore of great interest at the present time to explore new targeted therapeutic alternatives or adjuvant systems.

Non‐coding RNAs (ncRNAs) are classified based on their size as small ncRNA (< 200 nucleotides) and long ncRNA (lncRNA, > 200 nucleotides). The main representatives of small ncRNA are microRNAs (miRNAs), small interfering RNAs (siRNAs), Piwi‐interacting RNAs, and small nucleolar RNAs (snRNAs). There is growing evidence that deregulated ncRNA have an important function in the onset and progression of lung cancer (Berindan‐Neagoe *et al*., 2014; Catana *et al*., 2015), contributing to disease prognosis as well, and regulating the response to therapy (Braicu *et al*., 2014; Pan *et al*., 2017; Volinia *et al*., 2006).

MiRNA are short non‐coding transcripts approximately 22 nucleotides in length (Braicu *et al*., 2014; Calin and Croce, 2006; Redis *et al*., 2012; Strmsek and Kunej, 2015). By directly binding RNA from the messenger RNA and ncRNA categories, the function of a wide range of genes can be regulated through degradation of the RNA or inhibition of the translational processes (Braicu *et al*., 2015; Catana *et al*., 2015, 2017; Cipolla *et al*., 2018; Irimie *et al*., 2017a). An essential mechanistic feature of these transcripts relates to the partial complementarity to their target genes (Cipolla *et al*., 2018); therefore, a miRNA transcript can target multiple RNAs and a specific RNA can be regulated by several miRNAs (Berindan‐Neagoe and Calin, 2014; Berindan‐Neagoe *et al*., 2017; Braicu *et al*., 2014; Calin and Croce, 2006; Pop‐Bica *et al*., 2017; Sonea *et al*., 2018). MiRNAs have significant roles in all fundamental biological processes (cell differentiation or proliferation, apoptosis, cell cycle progression, invasion/distant metastasis and immune responses) (Eastlack and Alahari, 2015; Munker and Calin, 2011).

Alterations in miRNA expression levels are related to cancer pathogenesis (Chira *et al*., 2018; Sevignani *et al*., 2007). Generally, the transcripts with a reduced expression level have a tumor role, whereas overexpressed transcripts support oncogenesis (Berindan‐Neagoe *et al*., 2014; Catana *et al*., 2015; Irimie *et al*., 2017a; Munker and Calin, 2011). Moreover, due to their high stability, miRNAs can be found in different biological fluids either as free circulating molecules or incorporated in extracellular vesicles (e.g. exosomes). Variations of miRNAs levels in liquid biopsies are important minimally invasive diagnostic/prognostic tools and also therapeutic targets (e.g. exosome depletion; Gulei *et al*., 2018b; Pop‐Bica *et al*., 2018).

Currently, an increasing number of studies focus on experimental modulation of some miRNAs that are altered in different tumors to restore their normal expression level (miRNA inhibition or replacement; Berindan‐Neagoe *et al*., 2014; Braicu *et al*., 2014; Munker and Calin, 2011; Redis *et al*., 2012; Shah *et al*., 2016). In this review, we present an outline of recent studies on common and specific functions of miR‐181a and miR‐181b in lung cancers. A particular focus is on understanding the role of miR‐181a/b in lung cancer biology in order to facilitate the development of novel therapies based on miRNA modulation.

**Figure 1:**
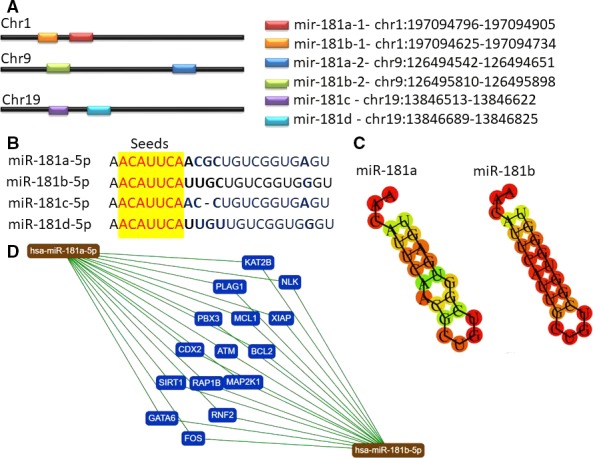
Localization, sequence and targets of the miR‐181 family members. (A) Chromosomal location of the members of the miR‐181 family and their sequence; genomic localization of miR‐181 family members was done using UCSC genome browser (https://genome.ucsc.edu). (B) Mir‐181 sequences containing the seed region (yellow) that is common for all transcripts. (C) MiR‐181a and miR‐181b common validated target genes according to miRtargetLink human database (https://ccb-web.cs.uni-saarland.de/mirtargetlink/).

The miR‐181 family is highly conserved in different species (Yang *et al*., 2014). This family contains four mature members, of which miR‐181a and miR‐181b are located on chromosomes 1 and 9, and miR‐181c and miR‐181d are clustered on chromosome 19 (Yang *et al*., 2017). As a consequence of genome duplication, miRNA‐181a as well as miR‐181b, have duplicate copies in the human genome, and can for this reason be derived from different precursors (Fig. 1).

miR‐181a and miR‐181b play diverse roles in regulating key aspects of cellular growth, development, angiogenesis, invasion, and metastasis in a wide range of solid tumors, including lung cancers (Xu *et al*., 2015). In this malignancy, the expression levels of miR‐181a and miR‐181b are decreased, indicating that depletion of these transcripts may facilitate lung tumorigenesis or disease progression, and activate drug resistance mechanisms (Cao *et al*., 2017; Cipolla *et al*., 2018; Liu *et al*., 2016; Shukla, 2018; Yang *et al*., 2013a). The downregulated profile of miR‐181a/b in cancer can be related to the methylation status, as proven in colorectal cancer (Shi *et al*., 2018), but no information regarding possible epigenetic regulation of the other two transcripts (miR‐181c/d) exists at this moment. Most of the research on lung cancer has centered on miR‐181a/b, probably with the expectation that the other members (miR‐181c and miR‐181d) have similar functions due to their identical seed sequences.

## 2 miR‐181a/b expression levels in lung cancer

miR‐181a/b are associated with a wide range of tumor and non‐tumor pathologies (metabolic disorders, neurodegenerative or infectious diseases, cardiovascular pathologies) (An *et al*., 2017; Sun *et al*., 2014a). These two transcripts can have dual roles, depending on different target genes or their mutational status (Seoudi *et al*., 2012). Overexpression was observed in breast (Bisso *et al*., 2013; Liu *et al*., 2017), ovarian (Lee *et al*., 2012; Li *et al*., 2016b; Parikh *et al*., 2014; Xia and Gao, 2014) and cervical cancer (Chen *et al*., 2014; Ke *et al*., 2013; Xu *et al*., 2016), whereas miR‐181a/b are generally downregulated in lung cancer (Cao *et al*., 2017; Cinegaglia *et al*., 2016; Huang *et al*., 2015; Liu *et al*., 2016; Ma *et al*., 2015; Tian *et al*., 2016; Wang *et al*., 2015a; Yang *et al*., 2013a) and glioblastoma (Ayala‐Ortega *et al*., 2016; He *et al*., 2016; Lakomy *et al*., 2011; Shi *et al*., 2008; Slaby *et al*., 2010; Sun *et al*., 2014b; Zhang *et al*., 2012; Zhi *et al*., 2014). Moreover, the expression levels of these transcripts are often cell type‐specific and, in some cases, transitory or consistent with the degree of cell differentiation (Chu *et al*., 2015; Zhang *et al*., 2017b).

The expression levels of these two transcripts in lung cancer are related to clinico‐pathological characteristics (Table 1) (Gao *et al*., 2010; Ma *et al*., 2015). Decreased expression levels were confirmed in a meta‐analysis study on NSCLC, further correlated with patient survival rate (Pop‐Bica *et al*., 2018). miR‐181a/b are associated not only with an unfavorable survival but also with TNM staging (Gao *et al*., 2010; Liu *et al*., 2016; Wang *et al*., 2015a; Yang *et al*., 2013a). MiR‐181b expression alone is related to overall survival (OS) and disease‐free survival (DFS) for NSCLC (Wang *et al*., 2015a; Yang *et al*., 2013a). Distant metastases are important factors in patient prognoses and are one of the main reasons for the failure of NSCLC treatment. MiR‐181a/b can be used as prognostic markers or as therapeutic targets for limitation of the spread of lung cancer, based on their direct regulation of metastasis (Wang *et al*., 2015a; Yang *et al*., 2013a).

**Table 1:** Summary of the relative expression of miR‐181a and miR‐181b in tissue and other biological specimens from patients diagnosed with lung cancer. N, normal tissue; T, tumor tissue; FC, fold change; ↓, downregulation; ↑, upregulation; ns, not statistically significant; AUC, area under the curve for the ROC (receiver operating characteristic)

Type of lung cancer	Expression level	Biological specimens and approach used for evaluation	Relevant finding of the study	Reference
NSCLC	↓miR‐181a	8 paired samples for microarray; 47 matched paired samples for qRT‐PCR	Microarray data FC: 0.42; qRT‐PCR FC: 0.54; a correlation with low miR‐181a, high clinical stage and lymph node positive leads to poor prognosis of NSCLC	Gao *et al*. (2010)
NSCLC	↓miR‐181b	35 patients with NSCLC and 24 normal tissues	↓miR‐181a in T versus N; FC: 0.3 ± 0.05, *P* ≤ 0.05	Cao *et al*. (2017)
NSCLC	↓miR‐181a	22 paired tissues	↓miR‐181a in T and cell lines; FC: 0.5 ± 0.2	Wang *et al*. (2017a)
NSCLC	↓miR‐181b	126 paired tissues	FC for miR‐181b N: 5.9 ± 0.9, T: 2.5 ± 0.7, *P* ≤ 0.01; ↓miR‐181b associated with unfavorable prognostic	Yang *et al*. (2013a)
NSCLC	↓miR‐181b	62 paired tissues	FC for miR‐181b N: 2 ± 1, T: 5.5 ± 0.5, *P* ≤ 0.01; ↓miR‐181b associated with TNM and metastases, inhibits metastasis by downregulation of HMGB1	Liu *et al*. (2016)
NSCLC	↓miR‐181b	27 match paired tissues	↓miR‐181b in T: 0.2978 ± 0.03, N 1.202 ± 0.06	Huang *et al*. (2015)
NSCLC	↓miR‐181b	20 NSCLC patients sensitive to therapy; 18 NSCLC patients’ non‐responders to therapy	↓miR‐181b in resistant to therapy cases; FC 0.8 ± 2.2 for sensitive (*n* = 20); 4.1 ± 1.8 for resistant (*n* = 20)	Wang *et al*. (2015a)
Stage I NSCLC	↑miR‐181b	Profiling study: 46 patients stage I NSCLC and 42 healthy control; qRT‐PCR validation: 20 NSCLC stage I and 30 healthy control	↑miR‐181a upregulated NSCLC versus healthy controls; exosomal miR‐181a a specific biomarker for ADC	Jin *et al*. (2017)
Lung ADC	↓miR‐181a	miRNA‐Seq for 7 paired samples; qRT‐PCR in 22 LA and 12 normal lung tissues	miRNA‐Seq: logFC for miR‐181a‐1 T: −1.1444 (*P*‐value 0.0006); logFC for miR‐181a‐2 T: −1.1145 (*P*‐value 0.0002); qRT‐PCR: logFC for miR‐181a‐1: −0.862 (*P*‐value 0.140); logFC for miR‐181a: 0.862 (*P*‐value 0.140)	Cinegaglia *et al*. (2016)
LSCC	↑miR‐181b	Profiling NGS Illumina: 9 LSCC and 9 ADC paired samples; qRT‐PCR validation 18 paired tissue and plasma	↑miR‐181b‐5p upregulated in tissue and plasma LSCC	Tian *et al*. (2016)
LSCC	miR‐181a	23 paired LSC 102 male LSCC Patients plasma and 101 healthy controls; 16 LSCC plasma and 16 healthy controls plasma	↓ miR‐181a in T versus N ↑miR‐181a in LSCC plasma versus healthy controls, FC: 3.04, AUC: 0.731; ns in LSCC plasma versus healthy controls plasma exosomes	Shan *et al*. (2018)

Despite a general downregulated profile for MiR‐181a/b, some studies report a different trend. MiR‐181a was found upregulated in an NSCLC model of Gefitinib‐resistant cells when compared with the sensitive counterparts. The same pattern was observed in the plasma samples of patients with acquired Gefitinib resistance compared with the levels measured before Gefitinib treatment from the same patients. A negative correlation between miR‐181a and GAS7 was identified in NSCLC tumors; moreover, increased GAS7 expression is associated with improved patient survival. These data sustain the role of miR‐181a/GAS7 axis in controlling Gefitinib resistance, an axis that could become a therapeutic target in these patients (Ping *et al*., 2018).

In an integrative analysis focused on the altered miRNA pattern in lung cancer, the authors found that miR‐181a/b/c were all downregulated in ADC samples (Cinegaglia *et al*., 2016). An unpaired analysis of 17 lung ADC tumors and seven normal tissue samples identified 11 statistically significant, differentially expressed miRNA transcripts, including underexpressed miR‐181b‐1 and miR‐181b‐2. Meanwhile, paired sample analysis demonstrated 22 statistically significant miRNAs, eight transcripts with a reduced expression level (including miR‐181a‐1 and miR‐181a‐2), and 14 transcripts with an increased level (including miR‐181c). In a database comprising 1491 lung ADC and 441 normal tissues, 13 overexpressed transcripts were identified, including miR‐181b, miR‐181c, and three downregulated transcripts (miR‐181a, miR‐574, and miR‐1247; Cinegaglia *et al*., 2016).

Lung squamous cell carcinoma (LSCC) displays a differential expression level of miR‐181a between plasma and tissue; a miRNA pattern for male LSCC patients from the TCGA dataset revealed a downregulation of this transcript which was further validated in another patient cohort of 23 paired samples of LSCC. The same study showed an overexpression of miR‐181a in plasma, but when independently analyzing only the exosomal fraction, the results were not statistically significant (Shan *et al*., 2018). Contradictory data showed that miR‐181b is overexpressed in tumor tissue and plasma in LSCC patients in a profiling study on nine LSCC (paired samples) and nine ADC (paired samples), followed by a validation on 18 LSCC paired tissue and plasma samples (Tian *et al*., 2016). Such data can also be explained by the limited number of samples analyzed, indicating that larger studies are essential.

All in all, the expression/function of miR‐181a/b in lung cancer is not always consistent between studies, prompting the dual or context‐dependent role of these transcripts. In addition, there is growing evidence from clinical studies that these miRNAs can act as biomarkers, but it might be more relevant to consider not only their absolute expression levels but also the balance between the expression of miRNA and targeted genes.

## 3 miR‐181a and miR‐181b mediate lung cancer hallmarks

**Figure 2:**
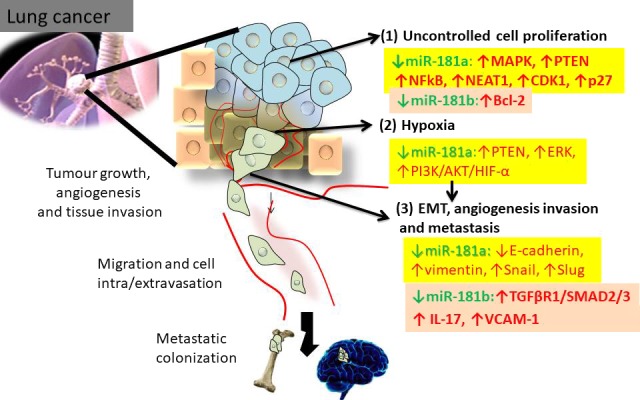
Relevant mechanistic insights connected with miR‐181a/b in lung cancer. (1) MiR‐181a/b target key genes involved in the regulation (1) of cell proliferation; (2) intra‐tumor hypoxia; (3) EMT, tumor angiogenesis, invasion, and distant metastasis.

Recent studies demonstrate that MiR‐181a/b are involved in the regulation of lung cancer hallmarks (Hanahan and Weinberg, 2011). MiR‐181a/b are downregulated in most of the studies and target important genes involved in the regulation of cell proliferation, evasion of growth suppression or resistance to cell death, as well as replicative immortality. These miRNAs also interfere with pathways involved in tumor angiogenesis, invasion, metastasis, and drug sensitivity/resistance in lung cancer (Fig. 2). Another less studied aspect is the connection with energy metabolism in lung cancer (Chu *et al*., 2015; Li *et al*., 2013).

### 3.1 miR‐181a/b inhibit proliferative signaling in lung cancer

miR‐181a/b are involved in mechanisms related to proliferation and growth signals (Shi *et al*., 2017). A549 cells transfected with miR‐181a‐5p mimic have a decreased cell proliferation and migration rate compared with the control counterparts, the effect being mediated in part by targeting of *K‐RAS* (Ma *et al*., 2015) and *MAPK* activity (He *et al*., 2015). Exposure of lung cancer cells (H226 and H460 cells) to interleukin (IL)‐17 decreases miR‐181a levels and upregulates *VCAM‐1* expression, a direct target. Administration of miR‐181a attenuates cell proliferation and migration rates, demonstrating the therapeutic potential of the *IL‐17*/miR‐181a/*VCAM‐1* axis (Wang *et al*., 2015a). Contradictory data were shown in a study where treatment of A549 cells with anti‐miR‐181a oligonucleotides (AMO‐miR‐181a) increased the apoptosis rate and lead to S‐phase cell (Fei *et al*., 2008).

### 3.2 miR‐181a/b target key genes involved in evasion of growth suppression or resistance to cell death, and replicative immortality

miR‐181b is downregulated in A549/cis (cisplatin resitant) compared with A549 cells, suppression that mediates drug‐resistant mechanisms and migratory features. Replacement strategies decreased cell proliferation, enhanced the sensitivity of the cells to cisplatin, and impaired the migratory phenotype in both *in vitro* and *in vivo* models. *Transforming growth factor receptor 1 (TGFβR1)* is a direct target of miR‐181b; moreover miR‐181b mimic administration decreased *c‐Myc* and *Cyclin D1* and upregulated *p27*, results that overlap with those obtained by siRNA‐*TGFβR1* transfection (Wang *et al*., 2015a). MiR‐181a contributes to cell cycle arrest by the upregulation of the cell cycle inhibitor *p27^Kip1^* (Galluzzi *et al*., 2010).

Restoration of miR‐181a expression is connected with inhibition of cyclin B1 and D1 expression in NSCLC cells and direct targeting of *CDK1* (Shi *et al*., 2017). MiR‐181a targets apoptotic genes such as *Bcl‐2* in acute lung injury (Li *et al*., 2016a) and also in lung cancer (Huang *et al*., 2015). MiR‐181a activates the apoptotic signaling cascade by a p53 tumor‐suppressor independent mechanism (Galluzzi *et al*., 2010).

### 3.3 miR‐181a/b are important regulators of angiogenesis, invasion, and metastasis

Hypoxia is a frequent event in malignant solid tumors and is further connected to the activation of angiogenesis (Choudhry and Harris, 2018). A hypoxic environment in lung cancer promotes invasion and metastasis through activation of MAPK signaling and macrophage polarization (Zhang *et al*., 2014), two mechanisms that are connected with miR‐181a/b expression (Bi *et al*., 2016; Wang *et al*., 2017a; Yang *et al*., 2013b; Zhang *et al*., 2013). An important aspect of metastasis consists in activation of epithelial‐to‐mesenchymal transition (EMT), which enables cells to migrate and populate secondary sites (Expósito‐Villén *et al*., 2018; Gulei *et al*., 2017, 2018a; Tudoran *et al*., 2012). In solid tumors, including lung cancer, transformed cells that undergo EMT lose epithelial features and acquire mesenchymal features (Expósito‐Villén *et al*., 2018; Zhang *et al*., 2017a). This phenotype undergoes self‐renewal and presents an increased capacity for adaptation to diverse environments, while favoring invasion and migration. TGFβ and its receptors (TGFβR1 and TGFβR2) have an important role not only in the regulation of cell fate (cell proliferation and apoptosis; Zhang *et al*., 2017a) but, more importantly, in the regulation of EMT, being concomitantly modulated by miR‐181a/b. MiR‐181a is present in breast cancer as a TGFβ‐regulated ‘metastamir’ (Taylor *et al*., 2013) that activates and promotes invasive and metastatic processes (Ionescu *et al*., 2014; Parikh *et al*., 2014). MiR‐181a is a promoter of TGFβ‐mediated EMT in ovarian cancer.

Studies showed a connection between miR‐181b with TGFβ signaling and PI3K/AKT signaling (Wang *et al*., 2015a). The miR‐181b/PI3K/AKT signaling pathway is a fundamental axis not only for the regulation of cell proliferation but also for the EMT and metastasis in lung cancer (Fumarola *et al*., 2014; Zhao *et al*., 2018).

### 3.4 miR‐181a/b has the capacity to avoid immune destruction

The tumor microenvironment (TME), consisting of stroma and extracellular matrix elements as well as immune cells, has an important role in lung cancer progression and invasion, migration, and metastasis (Quail and Joyce, 2013; Wang *et al*., 2017b). The presence of tumor‐associated macrophages under the immunosuppressive M2 phenotype disturbs the tumor microenvironment and sustains disease advancement (Guo *et al*., 2016; Zhang *et al*., 2013). MiR‐181a was observed to have a higher level in M2 than in M1 phenotype (Bi *et al*., 2016). MiR‐181a regulates the M2 macrophage‐mediated migration and invasion capacity of tumor cells (Bi *et al*., 2016). M2 macrophages infiltrate the tumor tissue, sustained by the release of cytokines/chemokines (Zhang *et al*., 2017a), with a possible localization in lung tumor hypoxic regions, where miR‐181a can play important roles (Zhang *et al*., 2014). MiR‐181a has the capacity to regulate the activity of CD8^+^ T cell influx, and the downregulation of multiple phosphatases by miR‐181a leads to a reduction in T cell receptor signaling. Therefore, miR‐181a is actively involved in the pro‐tumorigenic symbiotic role between tumor cells and tumor microenvironment effectors (Rupaimoole *et al*., 2016).

### 3.5 miR‐181a/b‐related therapeutic strategy in lung cancer

The main issue regarding miRNA therapy consists of the development of efficient delivery systems. The principal pharmaceutical formulations for miRNA delivery are liposomes, polymeric nanoparticles, and viral systems (Irimie *et al*., 2017b; Jurj *et al*., 2017; Tomuleasa *et al*., 2014). There are clinical safety concerns regarding viral delivery systems; among the non‐viral systems, the most promising are represented by liposomes (Chen *et al*., 2016; Yang, 2015).

Oncogenic miRNA are generally restored to their homeostatic level through different types of molecules: AMO (anti‐miRNA oligonucleotide) or antagomiR (Simonson and Das, 2015), locked nucleic acid (LNA) (Stein *et al*., 2010), miRNA sponges (Ebert and Sharp, 2010), and miRNA masks and circRNA (circular RNA) (Greene *et al*., 2017) (Table 2).

**Table 2:** The main characteristics of various systems used for miRNA therapy

Therapeutic strategy		Delivery system	Characteristic	Mechanism	Reference
miRNA inhibition therapy (miRNA with oncogenic role)	AMO (Anti‐miRNA oligonucleotides) or antagomiRs	Chemically modified for direct delivery; peptide liked delivery for receptor targeting	Short, synthetic, single‐stranded oligodeoxynucleotides	miRNA degradation by direct binding to target transcript and recycling the antagomir sequence	Rinaldi and Wood (2018), Simonson and Das (2015)
	LNA	Naked delivery	Monocatenare sequences, some modification for increasing the specificity	Inhibition of miRNA by direct binding to seed region	Stein *et al*. (2010)
	miRNA sponges or decoys	Viral construct encoding multiple miRNA‐binding sites downstream of a promoter	Single‐stranded 23 nt RNA molecules complementary to the targeted miRNA that have been modified to increase the stability of the RNA and protect it from degradation	Block miRNA role by inhibition the binding to their targets	Ebert and Sharp (2010)
	miRNA masks	Liposomal delivery	Short single stranded RNA, 2′‐O‐methyl‐modified	To complement the miRISC binding sites in the 3′ UTR of the target mRNA; mRNA specificity	Wang *et al*. (2015b)
	circRNA (circular RNA)	Liposomal delivery	RNA structure from 3′ end of a downstream exon has been backspliced to the 5′ end of an upstream exon, displayed as continuous RNA loop	miRNA sponge inhibiting activity	Greene *et al*. (2017)
miRNA replacement therapy (miRNA with tumor suppressor role)	miRNA mimics	Liposomal delivery as mature miRNA, miRNA*‐*mimics, precursors	Mature miRNA sequence, can be chemical modified for increasing the stability	‘Mimic’ the role of endogenous miRNA, restore its loss of function as a tumor suppressor	Wang *et al*. (2015b)
	miRNA vectors	Viral construct encoding miRNA sequence	miRNA cassettes cloned into any site of different destination vectors designed	Restoration of the target miRNA by direct genomic integration	Chira *et al*. (2015), Wang *et al*. (2015b)

Regulation of lung cancer TME can be considered the ‘Achilles heel’ of therapy success (Mittal *et al*., 2016), where miR‐181a/b can be an important player. MiR‐181a can be involved in limitation of lung cancer spread, as it was demonstrated to have critical EMT regulatory targets (He *et al*., 2015). Therefore, miR‐181 might be used as a direct or indirect therapeutic target, not only for the effects on the tumor, but also to regulate the immune response effectors that favor EMT or interact with TME, thus affecting the response to therapy (Parikh *et al*., 2014; Ye *et al*., 2018).

MiR‐181a/b therapy in lung cancer generally implies replacement strategies (miR‐181a/b mimics or miR‐181a/b vectors) for restoring the normal expression level. An important number of studies use miR‐181a/b inhibitors for mechanistic studies or because of different expression signatures in cell‐specific contexts. Most of the studies investigating the biological significance of miR‐181a/b transcripts use commercial miR‐181a/b mimics, and, as delivery systems, the commercially available liposomes (e.g. Lipofectamine 2000) (Cao *et al*., 2017; Fei *et al*., 2008; Huang *et al*., 2015; Ma *et al*., 2015; Wang *et al*., 2015a).

Recently, there has been important progress in the development of nanoparticle‐based therapies which represent a promising approach (Anselmo and Mitragotri, 2016), as can be observed by the high number of recently preclinical studies using liposomal delivery for miR‐181a/b (Table 3). This remains to be validated in clinical trials.

**Table 3:** Cell culture‐based studies examples for the evaluation of the therapeutic efficacy in lung cancer. N/A, data not available; ↓, downregulated; ↑, upregulated; FC, fold change; NF‐κB, nuclear factor kappa beta

Pathology	*In vitro* systems	Therapeutic approaches/Delivery system	FC in lung cancer cells	Observation	Reference
NSCLC	H226 and H460	miR‐181a mimic and inhibitor (50 nM)/Lipofectamine 2000	N/A	miR‐181a overexpression reduce cell proliferation and migration via *VCAM‐1*,* NFκB and IL‐17*	Cao *et al*. (2017)
NSCLC	A549 cells	AMO‐miR‐181a/Lipofectamine 2000 (lipofectin: oligonucleotides 2.5 : 1)	N/A	AMO‐miR‐181a reduces cell proliferation by activation of apoptosis and S‐phase cell cycle arrest	Fei *et al*. (2008)
NSCLC	H23 and H522 cells	miR‐181b mimic/Luciferase reporter vector and Lipofectamine 2000	Relative luciferases intensity for 95 ± 10 NC, 38 ± 8, in H23 WT HMGB1 cell; 100 ± 10 NC, 90 ± 5, in H23 WT HMGB1 cell Relative luciferase intensity for 95 ± 10 NC, 56 ± 5, in H522 WT HMGB1 cell; 100 ± 10 NC, 90 ± 5, in H522 WT HMGB1 cell	*HMGB1* is a direct target gene of miR‐181b in NSCLC; miR‐181b inhibits cell migration and invasion in NSCLC	Liu *et al*. (2016)
NSCLC	A549	miR‐181a mimic/inhibitor (150 nm)/Luciferase reporter vector and Lipofectamine 2000	H23: 0.25 ± 0.01 H1299: 0.3 ± 0.01 A549: 0.6 ± 0.05; HCC827 0.7 ± 0.1 95‐D: 0.7 ± 0.2 and SPCA‐1 0.85 ± 0.1 versus bronchial epithelial cell line (BEAS‐2B)	miR‐181a mimic reduced cell proliferation and colony formation, cell migration; target *Kras*	Ma *et al*. (2015)
NSCLC	A549, A549/PTX and A549/cis	50 pmol of miR‐181a inhibitor mimic/Lipofectamine 2000	↑miR‐181a in A549/PTX: 16 ± 1 A549/cis: 16 ± 1 than A549: 1 ± 0.2	miR‐181a targets PTEN; miR‐181a inhibitor reduces cell migration, invasion and expression of EMT‐associated genes; miR‐181a inhibitor sensitizes cancer cells to chemotherapy	Li *et al*. (2015)
NSCLC^a^	A549, H226, H460, SW‐900, HBE	MiR‐181 mimic and inhibitor/Lipofectamine 2000	↓miR‐181 in A549, H460, H358, and H1299 was about 18.10, 10.85, 7.08, and 16.98%, versus normal human bronchial epithelial cell line HBE	miR‐181 mimic leads to the inhibition of cell proliferation, migration, and invasion and promotes cell apoptosis; miR‐181 targets *Bcl*‐2, being involved in apoptosis regulation	Huang *et al*. (2015)
NSCLC	A549, A549/cis and H1650	miR‐181 inhibitor/mimic and negative control/Lipofectamine 2000	↓miR‐181b in insensitive to therapy; Relative expression level in HBE: 70.39, A549/cis: 1 A549: 3.11, H1650: 5.94	miR‐181b enhances chemosensitivity of NSCLC cells to Cisplatin; miR‐181b attenuates migration and invasion, modulates EMT; TGFβR1 has a critical role in miR‐181b‐mediated cell growth, chemosensitivity to cisplatin and metastasis of NSCLC cells	Wang *et al*. (2015a)
NSCLC	NSCLC A549, H1650, H1975, and HCC827, HCT116cells	pre‐miR‐181a and anti‐miR‐181a/Oligofectamine, HiPerFect (GFP)‐Bax–coding plasmid	↓miR‐181 tumor cell lines ↑ in cisplatin resistant cells	Pre‐miR‐181a modulated mitochondrial/post‐mitochondrial steps of the intrinsic pathway of apoptosis and potentiate the effect of cisplatin, carboplatin and Oxaliplatin	Galluzzi *et al*. (2010)
NSCLC	A549, and A549/cis	Mature miR‐181a/b/c/d mimic (100 nm)/Lipofectamine 2000	↓miR‐181b in A549cis than A549	*Bcl‐2* is targeted by mature miR‐181s; miR‐181b modulates multidrug resistance by inhibiting Bcl‐2 and sensitizes cells to apoptosis	Zhu *et al*. (2010)
NSCLC	PC‐9, PC‐14 and PC‐9/cis and PC‐14/cis	miR‐181a mimic and inhibitor/RNAiMax	↑ miR‐181a/b/c/d in A549cis than A549	miR‐181 inhibition has minimal effects on resistance to therapy	Pouliot *et al*. (2013)
NSCLC	PC9, NSCLC cell line A549 and A549/cis	miR‐181a mimic and inhibitor/Lipofectamine 2000	↓miR‐181a in A549 (FC 0.5), A A549/cis (FC 0.2), A A549/PTX (FC 0.3), H299 (FC: 0.8), H299/cis (FC: 0.5), H299/PTX (FC: 0.6)	SNHG12 is ↑ and miR‐181a is ↓ in NSCLC tissues and cell lines; SNHG12 regulates MAPK/Slug pathway by sponging effect of miR‐181a	Wang *et al*. (2017a)
NSCLC	HBE, A549, A549/cis H1650, H1650	miR‐181a mimic and inhibitor/Lipofectamine 2000	HBE FC: 70.39, A549/cis FC: 1, A549 FC: 3.11, H1650 FC: 5.94	miR‐181b mimic reduced proliferation, enhanced chemosensitivity to cisplatin, attenuated migration and metastatic rate	Wang *et al*. (2015a)

a Not specified miRNA type.

## 4 Implication of miR‐181a/b in lung cancer drug resistance

Chemoresistance is frequently observed in most lung cancer subtypes (Li *et al*., 2015; Shanker *et al*., 2010). Deciphering the molecular basis of drug resistance will lead to more effective treatments (Shanker *et al*., 2010). Knowledge‐based improvements in the field of predictive biomarkers for personalized treatment that rely on combining novel agents focused on resistance pathways with standard chemotherapy, might lead to the development of therapeutic designs capable of overcoming chemoresistance. The restoration of the miR‐181a/b expression level can be considered an important adjuvant strategy in lung cancer therapy for the prevention of drug resistance, as demonstrated by the large number of translational studies (Li *et al*., 2015, 2016a; Niu *et al*., 2016; Wang *et al*., 2017a). Nevertheless, we should not underestimate the important role of the immune system effectors and other host cells within the organism microenvironment.

Despite the increased interest in non‐cytotoxic targeted agents, systemic chemotherapy (Docetaxel, Gemcitabine, Irinotecan, Paclitaxel, Pemetrexed, and Vinorelbine) along with some targeted agents (Bevacizumab, Erlotinib, and Gefitinib) remain the pillar of therapy for lung cancer (Kim, 2016). Recent studies showed an increased use and clinical activity for the immune checkpoint inhibitors in lung cancer therapy. Understanding the regulatory mechanisms of PD‐L1 has become one of the biggest challenges for further improving therapeutic efficacy (Smolle *et al*., 2017). MiR‐181a  targets the ubiquitin ligases *Cbl‐b* and *c‐Cbl*; these two factors are negatively correlated with PD‐L1 expression in tissue samples from NSCLC patients and are proved to inhibit PD‐L1 *in vitro* through inactivation of ERK, STAT, and AKT signaling (Wang *et al*., 2018). Restoration of miR‐181a along with anti‐PD‐1/PD‐L1 might potentiate the therapeutic efficacy in lung cancer (Smolle *et al*., 2017), this being a research direction for future investigations.

miR‐181b overexpression inhibits cell proliferation and increases the sensitivity of lung cancer cells to DDP, attenuating at the same time the metastatic characteristics of the NSCLC cells (Wang *et al*., 2015a). The activity of miR‐181a/b is complex and is regulated at diverse levels (Lang *et al*., 2017; Wang *et al*., 2017a). MiR‐181a is sponged by small nucleolar RNA host gene 12 (*SNHG12*), a lncRNA that is overexpressed in lung cancer and inversely correlated with miR‐181a levels. Silencing of the lncRNA resulted in increased expression of the miRNA together with suppression of *MAPK1* and *MAP2K1* mediated by the high levels of miR‐181a achieved. The experimentally modified regulatory axis has further effects upon increased drug‐induced apoptosis in lung cancer cells (Fu *et al*., 2018; Wang *et al*., 2017a).

MicroRNA are involved in signal transduction, connected with drug metabolism and resistance, with potential use in personalized therapy (Gong *et al*., 2014). MiR‐181a and miR‐181b can also be used to increase the sensitivity to chemotherapeutic agents in lung cancer. MiR‐181a/*PTEN* is a novel regulatory circuit that mediates EMT in drug‐resistant lung ADC cells (Li *et al*., 2015). Lung cancer cells with acquired resistance to paclitaxel and cisplatin present a differential profile for miR‐181a with respect to their sensitive counterparts. Concomitantly, *PTEN* is reduced in these drug‐resistant models and is validated as a direct target of miR‐181a. Modulation of miR‐181a may become a promising strategy to prevent resistance to the main chemotherapeutics, in spite of the fact that some studies show minimal effects of miR‐181a/b on cisplatin‐resistant cells (Li *et al*., 2015; Pouliot *et al*., 2013). It is important to underline the necessity for further studies to show whether miR‐181 family members are capable of preventing the activation of chemoresistance mechanisms. The importance of the TME in chemotherapy efficiency is limited to *in vitro* studies or to immunocompromised mice models, decreasing the true translational value of miR‐181a modulation.

One major mechanism related to drug resistance is the malfunctioning of apoptosis pathways and the activation of complex compensatory pathways (Braicu *et al*., 2013, 2014; Pileczki *et al*., 2012). Studies link the overexpression of the proapoptotic gene *Bcl‐2* with the downregulated profile of miR‐181b in multi‐drug‐resistant lung cancer cells; after validation of direct inhibition of miR‐181b on *Bcl‐2*, replacement therapies showed significant improvement in terms of cell sensitivity to chemotherapeutic agents (Zhu *et al*., 2010). The transfection with mimic sequences showed a significant reduction in cell proliferation in A549/cis cells treated with vincristine, 5‐fluorouracil, cisplatin, and etoposide, but not mitomycin C (Zhu *et al*., 2010). Another study focused on assessing the impact of miR‐181b in modulating chemoresistance, evaluated the expression of the transcript in HBE cells (normal lung epithelial cell line), as well as in A549, H1650, and A549/DDP lung cancer cell lines (Wang *et al*., 2015a). qRT‐PCR showed that miR‐181b is downregulated in A549/DDP cells compared with sensitive cancer cells and significantly increased in HBE normal cell lines compared with all three malignant models (Wang *et al*., 2015a). Functional studies of miR‐181b upregulation showed that the proliferation and migration rates was significantly decreased and sensitivity to the treatment was restored. Moreover, *TGFβR1* was validated as a direct target of miR‐181b, where siRNA inhibition of the receptor gene showed similar results as miR‐181b overexpression (Wang *et al*., 2015a).

miR‐181a is related to Gefitinib resistance in lung cancer through an increased expression profile compared with the sensitive models and direct targeting of GAS7; GAS7 is involved in the regulation of AKT/ERK pathways and EMT markers and is downregulated in plasma from Gefitinib‐resistant patients (Ping *et al*., 2018). These findings indicate that restoring the expression of miR‐181a/b in lung cancer may play a critical role in fighting chemoresistance.

miR‐181a/b have the capacity to modulate drug resistance mechanisms in cancer cell lines, but the data remain inconsistent and need to be validated further in animal models. Taken together, all preclinical studies underline the therapeutic potential of these transcripts in the regulation of drug resistance. To be able to exploit these findings fully, it is mandatory to study this mechanism in the context of the complex TME.

## 5 Conclusions

Research performed in recent years demonstrates a wide range of novel functions for miR‐181a and miR‐181b in lung cancer. These studies reveal an important number of mechanisms that have clinical relevance but, at the same time, there are many issues related to the utility of miR‐181a/b in lung cancer management. One aim would be to build a global network that would integrate and interconnect the effects of all types of cells that constitute the TME with the mutational status of the genes that take part in the altered mechanisms. Deciphering this will lead to new and unexpected insights that will contribute to the development of novel and more efficient therapies for lung cancer.

miR‐181b is generally downregulated in lung cancer, and the reduced expression leads to an unfavorable prognosis in most of the cases. Therapeutic targeting of miR‐181a/b may be achieved at multiple levels, as shown by the preclinical studies, but at this moment there are no clinical trials of this. Additional studies are required to confirm the role of these two transcripts as biomarkers or therapeutic targets able to promote a less aggressive disease. MiR‐181a/b regulate structural and cellular elements involved in cell proliferation, as well as cell plasticity and adaptive programs that favor lung cancer invasion and migration.

The recently described role of miR‐181a/b in prevention of drug resistance by restoring the physiological expression levels, is an example of a sophisticated mechanism of action, which further underlines the fact that the expression level of a miRNA is not enough to propose it as a biomarker or therapeutic target. Consequently, this needs to be supported by additional functional studies of a specific phenotype able to prevent resistance to therapy or limit the spread of lung cancer. The biological role of miR‐181a/b needs to be studied in more detail, and the studies should not be limited to a simple exploration of the expression level but should be associated with complex characterization of genomic, transcriptomic, or epigenetic portraits.

## Author contributions

CB wrote the manuscript, DG and LR acquired the data, RC and EK drafted and revised the manuscript, AJ prepared the figures. GAC and IB‐N designed the project. All the authors contributed to the writing of the manuscript and approved the final version.

## Conflicts of interest

The authors have no conflicts of interest to declare.
